# The Norwegian CDI-III as an assessment tool for lexical and grammatical development in preschoolers

**DOI:** 10.3389/fpsyg.2023.1175658

**Published:** 2023-07-24

**Authors:** Elisabeth Holm, Pernille Bonnevie Hansen, Anna Sara H. Romøren, Nina Gram Garmann

**Affiliations:** ^1^Department of Early Childhood Education, Oslo Metropolitan University, Oslo, Norway; ^2^Department of Scandinavian Languages and Literature, Inland Norway University of Applied Sciences, Hamar, Norway

**Keywords:** language acquisition, parental report, CDI, vocabulary, assessment, preschoolers, item difficulty

## Abstract

Parental report instruments are a non-invasive way to assess children’s language development and have proved to give both valid and reliable results when used with children under the age of 2;6 (and in some cases up to 3). In this study we examine the newly developed Norwegian edition of a language assessment tool for older preschoolers: *MacArthur-Bates Communicative Development Inventory III* (CDI-III), investigating whether this parental report tool can be used for assessing the language of monolingual Norwegian-speaking children between 2;6 and 4 years. NCDI-III results for 100 children between 2;6 and 4.0 are presented. All sections were significantly intercorrelated. All sections except *Pronunciation* showed growth with age. Internal consistency was measured both in terms of Cronbach’s alpha and corrected item-scale correlation, and the results are discussed considering features of item difficulty distribution. Methodological considerations are discussed, as well as implications relevant both for possible later revisions and for CDI-III adaptations to new languages.

## 1. Introduction

Valid and reliable language assessment tools can be useful for a number of purposes, both for researchers and practitioners. The many language adaptations of the parental report tool *MacArthur-Bates Communicative Development Inventories* (CDI) have been used for, e.g., research on children’s language acquisition in specific languages (e.g., Wehberg et al., 2008; Kristoffersen et al., 2012) and cross-linguistic research about children’s language development (e.g., Bleses et al., 2008; Frank et al., 2021). Other examples of research are the use of CDIs to investigate how the language development of children with conditions such as cleft lip and palate, autism, impaired hearing, or language delay may differ from that of their typically developing peers (e.g., Scherer and D’Antonio, 1995; Charman et al., 2003). At the same time, CDIs are also used clinically or as screening tools by speech and language practitioners, child healthcare services and others. Some CDI adaptations have been validated with clinical use in mind (Heilmann et al., 2005; Thal et al., 2007; Urm and Tulviste, 2021).

The MacArthur-Bates CDI (Fenson et al., 2007) was constructed to capture reliable, precise and generalizable information about children’s early communicative development through a report form filled in by parents (Fenson et al., 1994). The tool is widely recognized as an effective, cost-efficient and valid method for assessing a range of communicative skills in young children, yielding reliable measures of early language development across languages (Law and Roy, 2008). Originally, there were two questionnaires, *CDI I Words and gestures* for children aged 8 to 20 months, and *CDI II Words and sentences* for children up to 30 months. The former comprises a section on early communicative development, including questions about gestures and imitation as well as a vocabulary checklist of about 300 words. In the latter, the vocabulary checklist is twice as long, and includes a list of grammar questions concerning overgeneralizations and sentence complexity as well as a question about the child’s three longest utterances. While some sections of the tool can be used to create compound scores, such as an estimated vocabulary size, the aim of its creators was to investigate language development in a broad sense, not to establish an overall measure of language development (Fenson et al., 1994).

Although the tool was meant to give an estimate of several aspects of children’s language skills, each section was not meant to give an exhaustive overview. Hence, the CDI vocabulary checklist, despite being quite extensive, was never meant to provide a full overview of any one child’s vocabulary, but rather to give an index of their vocabulary knowledge (Fenson et al., 1994). The introduction of shortforms (see, e.g., Jackson-Maldonado et al., 2013) further underlines this principle, as shorter versions of CDIs were developed to limit the workload involved in assessing children’s knowledge.

Combining CDI data from multiple languages, researchers have been able to study children’s language development across a wide variety of languages and societies. A recurring pattern is the great variation both in the pace of children’s language acquisition and in their routes to language learning (Frank et al., 2017). Another common pattern is a clear gender effect: Girls tend to outperform boys on productive vocabulary, whereas this pattern is less clear for word comprehension (Eriksson et al., 2012; Frank et al., 2021). Effects of sociodemographic factors on vocabulary have also been described, but these effects vary between languages (Frank et al., 2021).

### 1.1. The development of CDI-IIIs for 3–4-year-olds

The first attempts at expanding the CDI methodology beyond 2;6 years were made for American English by researchers in the US (Dionne et al., 2003; Feldman et al., 2005; Fenson et al., 2007). They used the same categories of words as in CDI-II, keeping some of the CDI-II words and adding some new ones. This type of CDI-III forms for children up to 4 have subsequently been developed for (Mexican) Spanish (Pilot INV-III, Guiberson, 2008), Basque (Ezeizabarrena et al., 2013; Garcia et al., 2014), and Hungarian (Kas et al., 2022). Whereas CDI I and IIs have been found to represent a valid measure of children’s language development of younger children, results from this type of CDI-IIIs have been more mixed, particularly concerning vocabulary (Eriksson, 2017). This was also the case for a previous version of the Norwegian CDI-III, where ceiling effects were found for the vocabulary section, and grammar items were found not to correlate with vocabulary (Sunde et al., 2014).

The requirements of the construction, validation and standardization of any assessment tool depend to a certain extent on what the main purpose of the instrument is intended to be: If it aims to capture the range of variation in language acquisition among children in its target population, the scales must have good discriminatory power across the full range in that population. As assessing vocabulary in older children necessarily entails capturing a very small subset of the children’s actual vocabulary (compared to the case for very young children), selecting the appropriate set of words is by no means trivial. To cover the intended age ranges of the CDI-IIIs, the instruments need to include both words that are ‘easy’ enough to distinguish between children with a relatively small vocabulary and words that are ‘sophisticated’ enough to distinguish between children with a fairly large vocabulary. That is, they must contain an assembly of items that are easier, items that are harder and items that are somewhere in the middle. At the same time, the instrument should preferably not be too long and extensive to administer.

To handle this problem, Eriksson (2017) proposed that rather than selecting words from a wide range of semantic domains, as do the previous *MacArthur-Bates CDI* instruments, choosing a smaller set of pre-defined themes based on developmental literature and being relatively exhaustive within these topics might be a better approach (2017, p. 648). The Vocabulary section of the Swedish CDI-III adaptation (SCDI-III) is hence built around four domains believed to be central for children in general and where their vocabularies typically expand during the preschool years. These themes are *food words*, *body words*, *mental words* and *emotion words*. The *food* theme is selected because food is an essential part of life and words related to food are usually found in children’s early vocabulary. Most of the words about food in the SCDI-III are verbs linked to cooking. The *body* words are selected to include both external and internal body parts, words for health conditions and body functions. Eriksson argues that children often begin to acquire words for external body parts during their second year of life, while words for internal organs, invisible to the child, are more demanding and are acquired at a slow pace. Being more abstract, *mental words* (words about thoughts) and *emotion words* (words about feelings) tend to be acquired from around the age of three, and children tend to acquire them at a slow pace during the preschool years.

The SCDI-III also introduced another invention: a *Metalinguistic awareness* section, included because these are skills generally acquired between 3 and 4 years while also being known to predict literacy (Eriksson, 2017, p. 648).

After conducting a validation and norming study, Eriksson (2017, p. 652) concluded that the structure of the Swedish version of the CDI-III could “well be integrated in similar instruments designed for other languages and cultures,” and CDI-III adaptations based on the Swedish version have since been developed for several new languages: Norwegian (Garmann et al., 2019), Polish, Finnish (Stolt, 2023), Estonian (Tulviste and Schults, 2020), (Mexican) Spanish (Jackson-Maldonado et al., 2022), and most recently Ukrainian. Both the Estonian version and the Norwegian one are based directly on the Swedish approach and mostly use the same categories as Eriksson (2017), while still being adaptations, not translations. Table 1 gives a brief comparison of the structures of the American English CDI-III and the Swedish, Estonian, Finnish and Norwegian adaptations. Below, we will present the structures of the Norwegian NCDI-III and the Estonian ECDI-III with the Swedish SCDI-III as a starting point.

**Table 1:** Comparison of the number of items and score ranges (in parentheses) for the subscales of five CDI-III adaptations.

CDI-III adaptation	US English	Swedish	Estonian	Finnish	Norwegian
General communication	N/A^a^	1 (0–6)	1 (0–6)	6 (0–6)	1 (0–6)
Vocabulary	100 (0–12)	100 (0–100)	101 (0–101)	100 (0–100)	100 (0–100)
Grammar/syntax^b^	12 (0–12)	10 + 8 (0–36)	7 (0–14) + 10 (0–20)	8 (0–16) + 10 (0–20)^c^	8 (0–24) + 10 (0–40)
Metalinguistic awareness	N/A	7 (0–7)	3 (0–3) + 4 (0–4)	7 (0–7)	7 (0–7)
Pronunciation/phonology	N/A	1 item (0–2)	6 (0–7)	6 (0–7)^c^	2 (0–5)
Semantics/comprehension	12 (0–12)	N/A	N/A	N/A	N/A

^a^In the original US English CDI-III form, Fenson et al. (2007) asked whether the child ‘is combining words yet’. If not, assessment was aborted after the vocabulary checklist. This question did not contribute to any scale. ^b^As discussed in sections 1.1.1–1.1.3, Eriksson (2017) combines his two grammar/syntax scales into one Swedish grammar score, while Tulviste and Schults (2020) and the current paper treat them as two separate scales for Estonian and Norwegian, respectively. ^c^Stolt (2023) combines the two Finnish grammar scales *Morphology* and *Language complexity* with *Phonology* to a *Language structures* score (range: 0–43).

#### 1.1.1. The structure of the Swedish CDI-III

The SCDI-III consists of sections asking about the child’s general level of communication, vocabulary, grammar, metalinguistic awareness, and pronunciation. The first section, *General level of communication*, consists of only one item: Parents are presented with a list of six alternative descriptions and asked to check the one that is true for their child. The alternatives range from ‘My child does not speak yet’ to ‘My child often speaks in long sentences, like […]’. This section has a major ceiling effect and serves as a ‘filter’: If the parent indicates that the child does not speak, or is impossible to understand, they are not asked to answer any further questions (Eriksson, 2017, p. 649).

Next, there is a vocabulary section consisting of 100 words chosen from words belonging to four semantic domains: *food words* (16 items), *body words* (26 items), *mental words* (30 items), and *emotion words* (28 items). Parents are requested to indicate the words that they have heard the child say.

Two sections both address grammatical complexity: In the *Language complexity* section, respondents are asked to indicate which of two example sentences are more similar to the way their child speaks now. Each of the 10 items consists of one ‘simple’ and one ‘complex’ alternative, such as *Jag har choklad* (‘I’ve got chocolate’) versus *Jag har choklad på min glass* (‘I’ve got chocolate on my ice cream’). For each item the parent indicates on a three-level scale whether the child mostly speaks in line with the simple or the complex example: ‘always left’ – ‘equally often’ – ‘always right’. In the *Grammatical constructions* section, 8 items address various grammatical features, such as past tense morphology and passives, illustrated with examples and explanations. To reduce ceiling effects, Eriksson (2017, p. 648) decided to merge the two grammar scales, treating them as one broader *Syntax* scale with scores ranging from 0 to 36.

The SCDI-III *Pronunciation* section consists of only one item, asking the parent to compare the child’s speech to that of children of the same age, indicating whether the child sounds a little younger than their peers, similar to their peers, or a little more advanced than most of them. The final section consists of 7 items concerning *Metalinguistic awareness*, where respondents are asked to indicate ‘yes’ or ‘no’ to questions concerning various phenomena linked to later literacy – such as whether the child notices similar-sounding words, imitates the way people speak, shows interest in letters, or can write letters or words.

#### 1.1.2. The Estonian CDI-III

The Estonian CDI-III (ECDI-III) (Tulviste and Schults, 2020) has kept most of the structure in SCDI-III, with a few changes: The ECDI-III has 7 questions about grammatical constructions where the Swedish form has 8, and the Estonian vocabulary section consists of 101 words instead of 100. Furthermore, the *Metalinguistic awareness* scale is divided into two subscales: *Phonological awareness* (3 items) and *Orthographic awareness* (4 items). Lastly, the *Pronunciation* scale contains 5 new yes/no questions in addition to the original comparison item. These new questions concern “pronunciation difficulties” that the child may have and whether strangers can understand what the child says (Tulviste and Schults, 2020, p. 71).

#### 1.1.3. The Norwegian CDI-III

The Norwegian CDI-III (NCDI-III), like the SCDI-III, consists of a 100 words vocabulary checklist in addition to sections with questions about the child’s general level of communication, grammatical structures, sentence complexity, pronunciation, and metalinguistic awareness. Each section has a similar structure to that of the SCDI-III, with a few exceptions: The NCDI-III has a 4-level scale for the *Grammatical constructions* items and a 5-level scale for the *Sentence constructions* items, where their Swedish counterparts both have 3-level Likert scales. The NCDI-III scores thus range between 0 and 24 for *Grammatical constructions* and between 0 and 40 for *Sentence complexity*. The NCDI-III *Pronunciation* section consists of two items, where the Swedish has only one. As in the SCDI-III, there is one item asking parents to evaluate the child’s speech relative to other children of the same age. In addition, like the Estonian version, the NCDI-III includes an item asking parents to indicate whether people who do not know the child have trouble understanding what the child says (4 levels: ‘no, never’ – yes, sometimes’ – yes, often’ – ‘yes, always’).

### 1.2. Validation studies of the new CDI-IIIs

The SCDI-III was normed and validated as an assessment tool to measure children’s language skills by analysing data from 1,134 children aged 2;6 years to 4.0 (Eriksson, 2017). The ECDI-III and the NCDI-III have both been subjected to smaller evaluation studies based on data from 3-year-olds: The ECDI-III using data from 100 Estonian parents of 3-year-olds (Tulviste and Schults, 2020), while the NCDI-III has been piloted on data from parents of 28 Norwegian 3-year-olds (Garmann et al., 2019). However, neither of the two have yet been evaluated for the whole target age span.

All of the published reports have included assessments of the scales’ internal consistency, the intercorrelation between scales, and how the scores may be related to demographic factors such as gender and parental education level. For the SCDI-III, Eriksson (2017) also presents age-based norms with percentile levels, as well as examining the dimensionality of each SCDI-III scale. Tulviste and Schults (2020) compare ECDI-III results from children with and without reported difficulties with language development, assessing the sensitivity and specificity of the instrument as a potential screening or diagnostic tool for children with language difficulties. Garmann et al. (2019) compare the NCDI-III reports from staff at the children’s Early childhood education and care (ECEC) centers with those from parents, to see if reports from ECEC teachers combined with those from parents can be a reliable way to assess the linguistic development of bilingual children. None of the three studies, however, examine the structure of the *Vocabulary* scale (or any of the other scales) in terms of how easy or difficult the various items are.

### 1.3. The current study

In this study, we examine psychometric and linguistic properties of the NCDI-III based on parental report data covering the full CDI-III age span (2;6–4 years) to answer the following research questions:

1. Do the NCDI-III scales capture growth with age for children aged 2;6 to 4;0 years?
2. Which other demographic factors predict the children’s scores?
3. Do the scales correlate with each other?
4. Is there internal consistency within each of the NCDI-III scales (*Vocabulary*, *Grammatical constructions*, *Sentence complexity* and *Metalinguistic awareness*)?
5. What is the pattern of difficulty distribution among the items in each scale of the NCDI-III, and in each thematic *Vocabulary* word group?

CDIs have been used for many purposes. While we do not rule out diagnostic use of the tool once norms are in place and the tool has been tried out also on clinical groups, our focus is in the current paper is descriptive. Based on previous research, we expect higher scores with age on the lexical, grammatical and metalinguistic subscales (question 1) and higher scores among girls than boys (question 2). Furthermore, we expect the strongest correlations between the two grammatical scales, and between these and the vocabulary scale (question 3). Concerning the items within each scale, we expect internal consistency within all scales, with the possible exception of the metalinguistic scale (question 4). None of the previous studies describe the dispersion of item difficulty within the CDI-III scales (question 5).

## 2. Methods and materials

### 2.1. Data

The data used in this paper were collected by student assistants. The participants were recruited via the students’ own networks and by contacting ECEC teacher students and ECEC centers, and to a certain degree the recruitment was cumulative, with participants recruiting their own acquaintances (Flygstad and Milder, 2017). The NCDI-III and background forms were administered online, and parents were asked to fill them out digitally at their leisure. Inclusion criteria were that children were to be ‘monolingual’ (i.e., no household members with other first language than Norwegian), and that parents or ECEC staff reported no concerns regarding the child’s language development. All participating children attended kindergarten. All the parents received written information about the project and signed consent forms. Methods for data collection and data processing were developed in line with guidelines from and evaluated by the Data Protection Services at Sikt – Norwegian Agency for Shared Services in Education and Research (formerly NSD – Norwegian Centre for Research Data).

In this paper, we analyze parent-reported NCDI-III data from three age groups: 2;6, 3, and 4 years (see Table 2 for information on number of children, age ranges and gender distributions). Two children were excluded from the data set: One because they did not belong to the age group from which the relevant data set was collected, the other because there seemed to be something wrong with the way the form was completed. The data from the 3-year-olds are also discussed in Garmann et al. (2019), and subsets of the collected data have been used in the student assistants’ MA theses.

**Table 2:** Age and gender distribution in the dataset.

Age group	Boys: Girls	Mean age^a^ (SD)	Median age (range)
2;6-year-olds (*n* = 36)	18:18	2;6.8 (45.1 days)	2;6.13 (2;2.27–2;8.23)
3-year-olds (*n* = 28)	12:16	2;11.24 (28.0 days)	2;11.23 (2;10.13–3;1.18)
4-years-olds (*n* = 36)	19:17	3;11.30 (27.3 days)	3;11.30 (3;10.24–4;2.2)
Total (*n* = 100)	49:51		

^a^Age measured in days has been divided by 30.4 to calculate age in months.

The background form showed that 34 of the 100 children had no siblings or younger siblings only, while 66 had older siblings or a twin. As for parental education level, there was a skewness toward several years of higher education. Of the 100 participating parents, only 2 reported primary school as their highest education, 14 had upper secondary education, while 3 had higher education of less than 3 years and 28 had 3 years of higher education. More than half the sample, 53 of the 100 parents, had *more than 3 years* of higher education. In comparison, as many as 52–55% of the general population between 20 and 49 years *have no* higher education, according to numbers from Statistics Norway (2022). Among those who do, about 2/3 have studied for 4 years or less [calculated from numbers provided by Statistics Norway (2022); Table 1].

Because parents with lower education levels were so few in our dataset, parental education was collapsed into two levels in the analyses, distinguishing only between parents who had completed at least 3 years of higher education (81 parents) and parents with lower levels of education (19 parents).

### 2.2. Analyses

Score distributions in the NCDI-III sections were calculated for each age group. In addition, regression models were used to investigate to what degree the variation in NCDI-III scores is predicted by age along with other demographic factors (gender, sibling status and parental education). There were minimum and/or maximum scores in most of the scales, and we see these as probable results of the scales’ boundedness rather than real limits of the constructs measured: values outside the scope of the measure will appear as instances of the minimum value (left-censoring) or the maximum value (right-censoring). In our statistical analyses, we therefore used Tobit regression models (Tobin, 1958), as this method is suitable to estimate the relationship between variables when the dependent variable is censored.

Tobit regression models were used to investigate possible associations between demographic factors and children’s NCDI-III scores for *Vocabulary*, *Sentence complexity*, *Grammatical constructions* and *Metalinguistic awareness*. Apart from age, the demographic factors that were investigated were gender, parental education level and sibling status. To check for possible interactions between age and gender, preliminary models were fitted with such interactions as a fifth predictor variable. No significant interaction was found for any of the scales, and we thus report models fitted without interaction. Age measured in days is the basis for the age variable and has been divided by 30.4 to calculate age in months. The parental education item in the questionnaire had five levels, but as the four lower levels were merged into one category, the parental education variable in the regression models is binary, and only distinguishes between parents with more than 3 years of higher education and parents with any lower levels of education.

Intercorrelations among the NCDI-III scales and sections were calculated using Pearson’s product–moment correlation and Spearman’s rank correlation rho, controlling that there were no large differences. In this paper we report Pearson’s correlation coefficients; a comparison with other correlation coefficients can be found in Supplementary Appendix.

Cronbach’s alpha is a common way to investigate a scale’s internal consistency and thereby its reliability. Internal consistency was calculated in terms of Cronbach’s alpha for all scales (*Vocabulary*, *Sentence complexity*, *Grammatical constructions*, and *Metalinguistic awareness*). As Cronbach’s alpha is known to be strongly affected by the length of the scales (cf. Streiner, 2003), we also used corrected item-scale-correlation (see DeVellis, 2017) as an additional measure of internal consistency, to further investigate the internal consistency of the scales by a measure that is independent of the number of items in each scale.

The item difficulty distribution of each scale was analyzed, and for the *Vocabulary* section, we also examined the item difficulty profile of each of the four thematic word groups. We calculated difficulty values for each item; first globally, based on the whole sample of participants, and then for each age group separately. The item difficulty profiles of the four thematic word groups were based on the item difficulty values from the full sample.

For the dichotomous items in NCDI-III’s *Vocabulary* and *Metalinguistic awareness* scales, item difficulty is reported in terms of proportion values (0.00–1.00), i.e., the proportion of participants indicating that their child had said the word or exhibited the characteristic asked about by the item in question. For non-dichotomous items such as those in the *Sentence complexity, Grammatical constructions* and *Pronunciation* sections, item difficulty is reported in terms of average response values. Mark that this makes the difficulty measure ‘inverse’, in the sense that a *low* proportion value or a *low* average response value indicate that an item is considered more difficult, as few children received high scores on those items. Correspondingly, items with *high* mean scores or proportion values are considered easy.

Statistical analyses were carried out in R version 4.1.0 (R Core Team, 2021) using RStudio version 1.4.1717 (RStudio Team, 2021). Tobit regressions were modeled using the *AER* package version 1.2–10 (Kleiber and Zeileis, 2008). Cronbach’s alpha and item-rest correlations in the NCDI-III scales were calculated using the *psych* package (Revelle, 2021). Density plot and violin plots of item difficulty distributions were made with *ggplot2* (Wickham, 2016). Data editing and other analyses were performed with *dplyr* version 1.0.7 (Wickham et al., 2021) and the *base* package.

## 3. Results

The results are reported in the order of the research questions they address. Growth with age is first reported as seen in isolation and by age group, before age measured in days is presented as part of regression models along with gender, sibling status and parental education level. Correlations between sections are then analyzed, before the internal consistency of each scale is reported both in terms of Cronbach’s alpha and in terms of corrected item-scale correlations. Finally, the item difficulty profile of each scale is described – first as calculated for the total sample (including calculations for each thematic word group in the *Vocabulary* scale), and then as calculated for each age group separately – followed by the relationship between item difficulty distribution and item-scale correlation.

### 3.1. Growth with age

All 4-year-olds, and a majority of the other children, reached a maximum score in the single-item *General level of communication* section. None of the children had minimum or maximum scores in the *Vocabulary* section, but in all other sections, there were one or more children with maximum scores. For *Sentence complexity* and *Metalinguistic awareness,* there were some children with zero-scores and some with maximum scores. All sections except *Pronunciation* show growth with age.

As Table 3 shows, there is growth with age in every section except for the *Pronunciation* section, where growth with age was only evident in one of the two items. This item asks whether or not strangers find it difficult to understand the child’s speech, and has a score range from 0 (‘yes, always’) to 3 (‘no, never’). Among the 2;6-year-olds, 5 children (13.9%) were reportedly *always* difficult to understand for strangers. The number was reduced to one child among the 3-year-olds and one among the 4-year-olds. Twenty six of the 2;6-year-olds (72.2%) were reported to be difficult to understand *sometimes*. This was the case for 17 (60.7%) of the 3-year-olds, and for 13 (36.1%) of the 4-year-olds. Only 5 of the 2;6-year-olds (13.9%) were *never* difficult to understand for strangers, while this was the case for 10 (35.5%) of the 3-year-olds, and a total of 22 of the 4-year-olds (61.1%). The answers to the second *Pronunciation* question, asking parents to compare their child’s speech to that of other children of the same age, did not show any growth with age; rather, parents of 2;6-year-olds were the most likely to judge their child’s speech as resembling that of ‘slightly older children’.

**Table 3:** Median scores and score ranges of NCDI-III for each age group and across age groups.

NCDI-III scale	2;6-year-olds	3-year-olds	4-year-olds	Total
General level of communication (0–5)	4.5 (3–5)	5 (3–5)	5 (5–5)	5 (3–5)
Vocabulary (0–100)	43 (15–74)	55 (24–90)	70.5 (44–93)	59 (15–93)
Sentence complexity (0–40)	14 (0–32)	19 (1–40)	25 (11–38)	20.5 (0–40)
Grammatical constructions (0–24)	12 (3–23)	16 (4–24)	19 (1–24)	16 (1–24)
Pronunciation (0–5)	4 (1–5)	3.5 (1–5)	4 (2–5)	4 (1–5)
Metalinguistic awareness (0–7)	2 (0–5)	3 (0–6)	5 (1–7)	3 (0–7)

### 3.2. NCDI-III results and demographic factors

**Figure 1:**
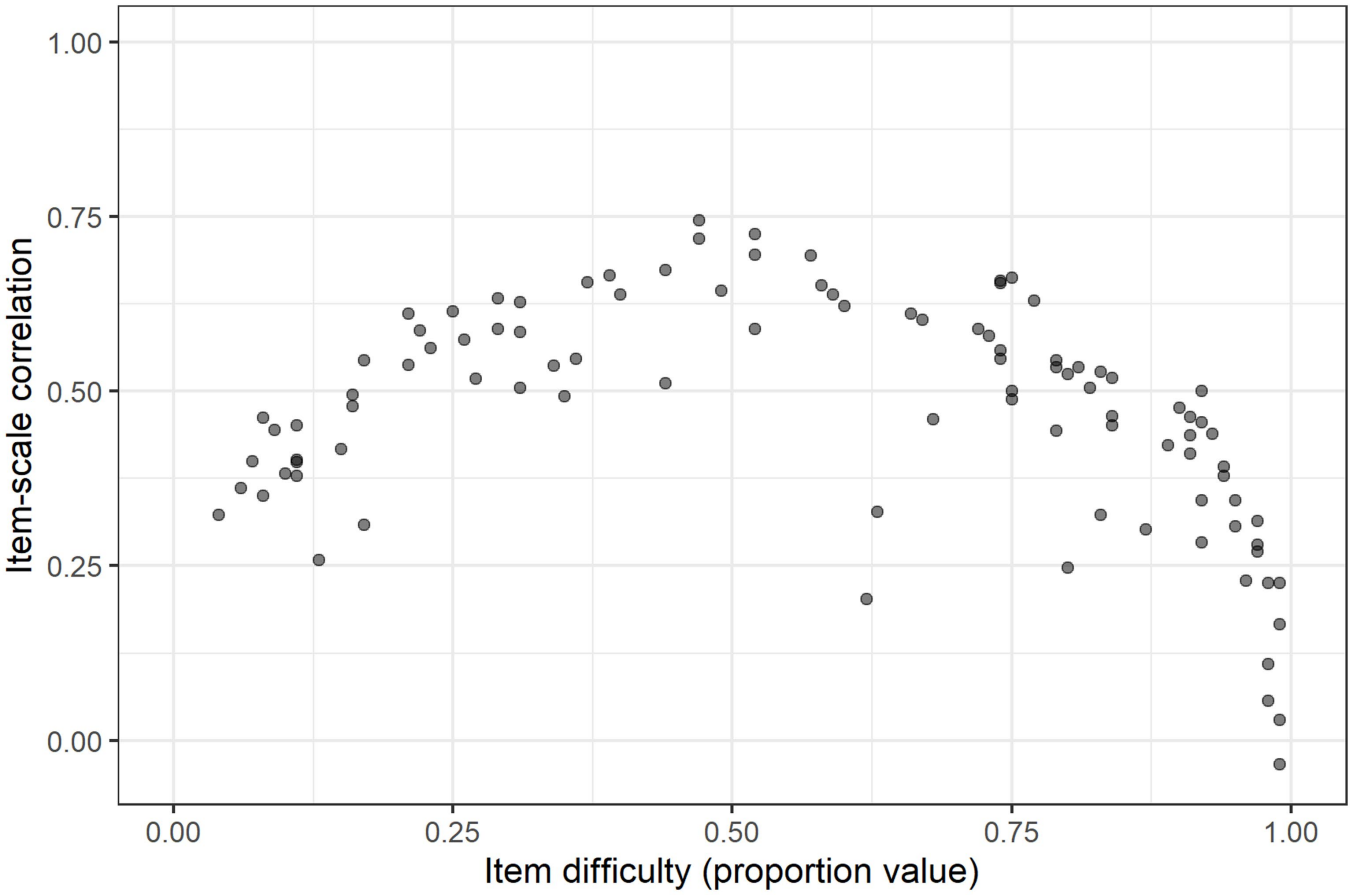
Scatterplot of vocabulary score by age and gender, with a fitted line for age (averaged between genders) based on the regression model in Table 4.

For *Vocabulary*, both age and gender were significant predictors in the final regression model [*W*(4) = 88.25, *p* < 0.001], while neither parental education nor the existence of older siblings had a significant effect on vocabulary results (see Table 4). As shown in Figure 1, vocabulary scores overall increased with age (1.58 words per month), and girls scored higher than boys (with a gender effect of 8.27 words).

**Table 4:** Regression model for overall Vocabulary score.

Variable	*B*	*SE*	*Β*	*p*
Constant	−7.399	8.443	−0.876	0.381
Education	−1.336	3.593	−0.372	0.710
Age (months)	1.585	0.180	8.803	<0.001
Gender	8.273	2.808	2.947	0.003
Sibling status	3.290	2.983	1.103	0.270
Log (scale)	2.630	0.071	37.187	<0.001

Scale range: 0–100. No left-censored or right-censored observations.

Table 5 shows the final Tobit regression model for *Sentence complexity*, where only age was a significant predictor [*W*(4) = 36.96, *p* < 0.001]. We observed a positive relationship between age and sentence complexity scores, with a sentence complexity increase of 0.65 per month, consistent with the pattern observed for vocabulary.

**Table 5:** Regression model for Sentence complexity.

Variable	*B*	*SE*	*Β*	*p*
Constant	−6.912	5.076	−1.362	0.173
Education	1.202	2.160	0.557	0.578
Age (months)	0.650	0.108	6.016	<0.001
Gender	1.874	1.686	1.112	0.266
Sibling status	0.264	1.788	0.147	0.884
Log (scale)	2.117	0.072	29.213	<0.001

Scale range: 0–40. 2 left-censored and 1 right-censored observation.

In the regression model for *Grammatical constructions* [*W*(4) = 22.44, *p* < 0.001], age was once more the only significant predictor (see Table 6). The score for grammatical constructions increased by 0.35 per month. That is, on average, every third month a new item is checked.

**Table 6:** Regression model for Grammatical constructions.

Variable	*B*	*SE*	*β*	*p*
Constant	1.466	3.555	0.412	0.680
Education	0.372	1.522	0.245	0.807
Age (months)	0.353	0.076	4.647	<0.001
Gender	1.246	1.186	1.051	0.293
Sibling status	−0.057	1.259	−0.045	0.964
Log (scale)	1.760	0.075	23.620	<0.001

Scale range: 0–24. 0 left-censored and 7 right-censored observations.

Table 7 presents the final regression model for *Metalinguistic awareness* [*W*(4) = 80.56, *p* < 0.001], parental education, age, sibling status and gender were all significant predictors. Age had a positive effect in the model, increasing the metalinguistic awareness score by 0.15 per month. So did parents’ education, with children of parents who had completed at least 3 years of higher education scoring 1.18 higher than children whose parents had not. Sibling status had a negative effect of 0.98, meaning that children without older siblings scored higher, and gender had a positive effect of 0.71, meaning that being a girl was associated with higher scores. (The scale range is 0–7, and there were 9 left-censored and 3 right-censored observations).

**Table 7:** Regression model for Metalinguistic awareness.

Variable	*B*	*SE*	*β*	*p*
Constant	−3.459	0.912	−3.791	<0.001
Education	1.174	0.389	3.021	0.003
Age (months)	0.153	0.019	7.935	<0.001
Gender	0.717	0.298	2.406	0.016
Sibling status	−0.978	0.316	−3.099	0.002
Log (scale)	0.372	0.077	4.798	<0.001

Scale range: 0–7. 9 left-censored and 3 right-censored observations.

For the NCDI-III sections *Pronunciation* and *General level of communication*, no regression model was fitted, as the former consists of only two questions and the latter has only one major ceiling effect. One of the two questions in the *Pronunciation* section furthermore differs from the rest of the tool by relating to development relative to the child’s age. Here, parents are asked to judge whether the child’s speech sounds most like that of younger children, children of the same age, or older children.

### 3.3. Correlation between scales

All sections correlated significantly with each other (*p* = 0.006 for the correlation between *Pronunciation* and *Metalinguistic awareness*, *p* < 0.001 for all others). As shown in Table 8 below, the strongest correlations were the ones between the *Vocabulary* section and the *Sentence complexity* (*r* = 0.70), and *Vocabulary* and *Grammatical constructions* (*r* = 0.67), as well as the two grammar scales’ correlation with each other (*r* = 0.66). The weakest correlations were found to be those of *Pronunciation* with *Vocabulary* (*r* = 0.36) and *Metalinguistic awareness* (*r* = 0.27) respectively.

**Table 8:** Correlations between the scales of the NCDI-III.

	Sent.comp.	Gram.constr.	Pronun.	Metaling.
Vocabulary	0.70***	0.67***	0.36***	0.52***
Sentence complexity		0.66***	0.42***	0.44***
Grammatical constructions			0.46***	0.45***
Pronunciation				0.27**

Pearson’s correlation coefficient. **p < 0.005; ***p < 0.001.

### 3.4. Internal consistency

As shown in Table 9, all scales had alpha scores above 0.65. The *Metalinguistic awareness* scale had a lower alpha score than the other scales; only 0.66 [though still ‘adequate’, in terms of Eriksson, 2017]. The two grammar scales both had high alpha levels: 0.92 and 0.89. The *Vocabulary* section had the highest alpha, at 0.97.

**Table 9:** Internal consistency of NCDI-III scales as measured by corrected item-scale correlations and Cronbach’s *α*.

	Item-scale correlations			α
	<0.30	0.30–0.50	>0.50	
Vocabulary (100)	14	37	49	0.97
Sentence complexity (10)	0	0	10	0.92
Grammatical constructions (8)	0	1	7	0.89
Metalinguistic awareness (7)	2	3	2	0.66

The number of items within each scale found within each correlation range. Total number of items for each scale listed in parenthesis.

Using corrected item-scale-correlations as a measure of internal consistency, the *Sentence complexity* and *Grammatical constructions* scales were still found to be highly consistent, but the *Vocabulary* section came out as far less consistent than suggested by the alpha score alone. Table 9 shows the distributions of corrected item-scale correlation coefficients for the items in each scale as well as each scale’s Cronbach’s alpha value.

### 3.5. Difficulty of items

**Figure 2:**
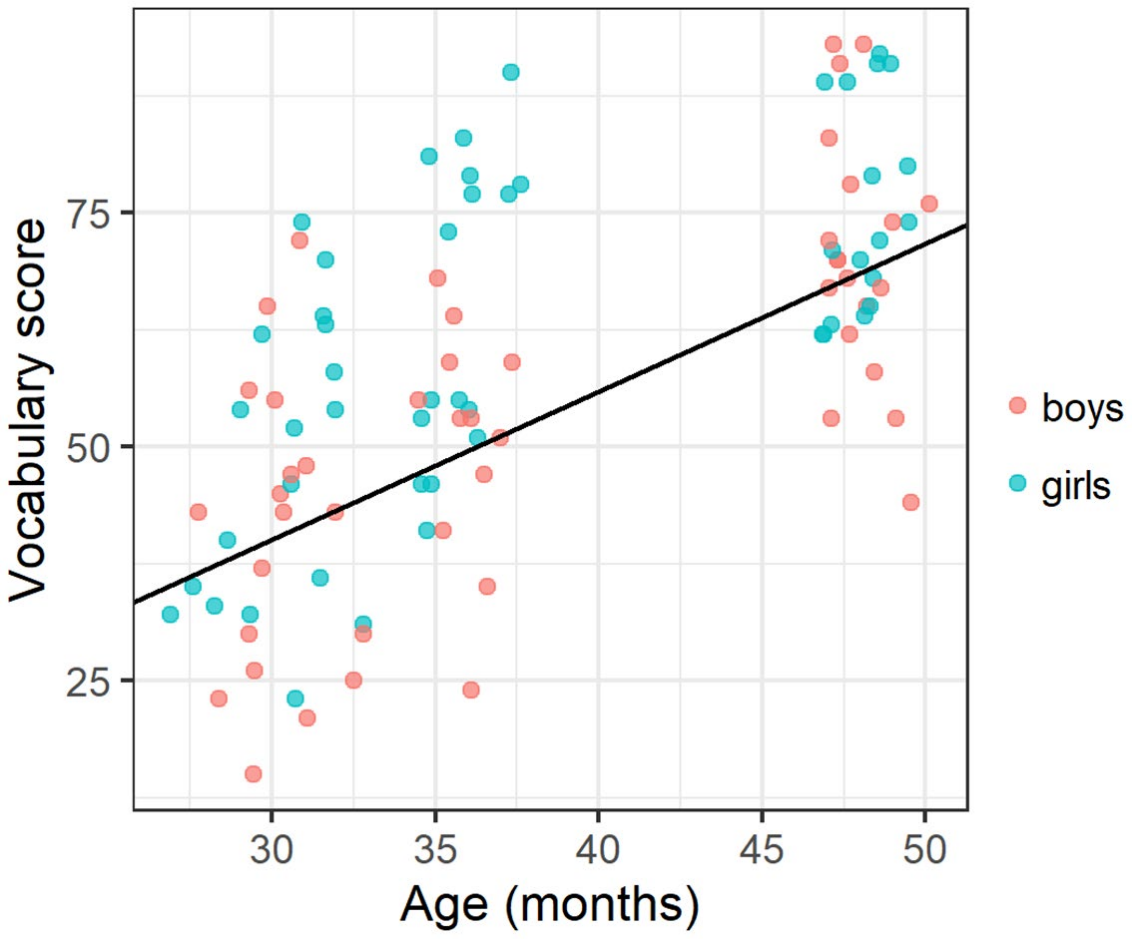
Density plot of the levels of item difficulty (proportion values) in the NCDI-III *Vocabulary* section (easier items have higher proportion values), with dots marking each observation. Bin width: 0.1.

All of the 100 words in the vocabulary section were checked at least once by one of the parents, but none of the words were checked by all parents. Median item difficulty was 0.66, range: 0.04–0.99. (Note that the item difficulty measure is ‘inverse’, meaning that higher values indicate ‘easier’ items and vice versa.) The variance in answers was, however, not symmetrically distributed, as shown in Figure 2.

Only 50 of the words had a difficulty in the range between 0.21 and 0.80, meaning that the other half of the words had difficulty levels closer to the ends of the range. Thirty of the 100 words were reported to be said by either 0–10% of the children or 91–100%. Most of these words were found in the easy end of the spectrum, with as many as 23 words reported to be said by 91% or more. Overall, the items of the *Vocabulary* section of the NCDI-III ranged from very easy to very difficult, but with fewer words near the middle range and more words near the poles.

**Figure 3:**
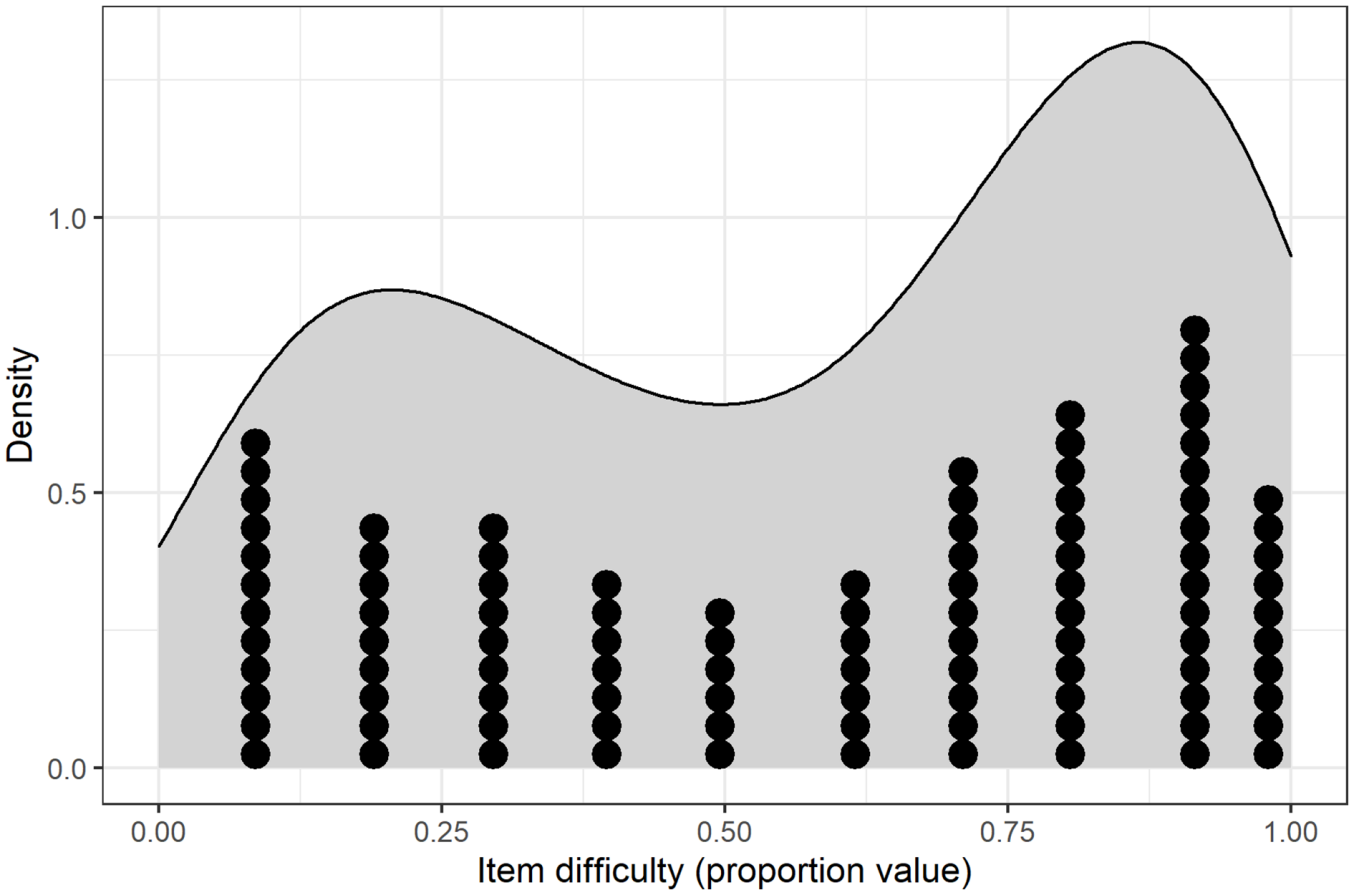
Violin plot of difficulty (proportion values) for each word in NCDI-III, by thematic word group (easier items have higher values). Bin width: 0.1.

As Figure 3 shows, the four thematic groups of words showed differing difficulty profiles in the NCDI-III results. In general, the *body words* and the *food words* tended to be easier than the words related to thoughts and feelings, with many of the body words concentrated near the easiest limit of the range. The *emotion words* and the *mental words* both had a more balanced distribution between easy and difficult words, but while the mental words showed an almost seamless gradient covering the whole difficulty span, the emotion words were divided into one set of easier and one set of more difficult words, with only one single word in the center of the scale. Thus, apart from the mental words, we found a split in all word groups, with easy and hard words, and few or none in between.

Among the items in the two grammar scales, there was less variance when it comes to levels of difficulty. The overall easiest item of the *Sentence complexity* scale (item response range: 0–4) had a mean response value of 3.14, and the other 9 items had mean values ranging from 1.56 to 2.29. The median item difficulty of the *Sentence complexity* scale was a mean response value of 1.89. In the *Grammatical constructions* section, the items (response range: 0–3) mainly covered the area from moderate to easy, with the most difficult item having a mean score of 1.34 and the easiest a mean score of 2.39 (median: 2.02).

The *Metalinguistic awareness* section’s dichotomous items, on the other hand, seemed to have more dispersed difficulty levels, especially among the older children. One item was exceptionally difficult (0.04) and the rest had proportion values ranging from 0.29 to 0.69 (median: 0.5). The most difficult item – asking whether the child can write some words on their own – was only checked for four children; all of them were 4-year-olds. As was to be expected, the distribution of item difficulty varied between the age groups. Among the 2;6-year-olds, the median *Vocabulary* item difficulty was 0.46 (range: 0–1), among the 3-year-olds it was 0.71 (range: 0–1) and among the 4-year-olds it was 0.86 (range: 0.08–1). The bimodal distribution with relatively few words in the middle of the scale observable in Figure 2 were apparent in all three age groups. For the 2;6-year-olds, there was a skewness toward words being difficult, whereas the opposite was true for the 3-and 4-year-olds.

In each age group, there were words that were reported to be said by every child, and among the 2;6-year-olds and among the 3-year-olds, there were also some words not marked as said by any child in the age group. Among the 2;6-year-olds (*N* = 36), 28 words were checked by 10% or less. On the other hand, 11 words were checked by more than 90%. Among the 3-year-olds (*N* = 28), 15 words were produced by 10% or less, and 26 words were reported to be produced by more than 90%. Among the 4-year-olds (*N* = 36), as many as 43 words were checked by more than 90%. In this age group, only 2 words were checked by 10% or less.

Among the items in the two grammar scales, the item difficulty variance was larger among the youngest children than the older ones. In the *Sentence complexity* scale, the gap between the easiest item and the rest seemed to shrink with age: Among the 2;6-year-olds, the easiest item had a mean response value of 2.86, while the other items’ values ranged between 0.83 and 1.83. Among the 3-year-olds, this item’s mean response value was 3.29, with the others ranging from 1.43 to 2.29, and among the 4-year-olds, the other items (2.03–2.92) had more or less caught up with it (3.31). The median item difficulty of the *Sentence complexity* scale increased from 1.21 among the 2;6-year-olds to 1.93 among the 3-year-olds and 2.54 among the 4-year-olds. In the *Grammatical constructions* section, the median grew from 1.5 among the 2;6-year-olds (range: 0.89–2.11) to 2.21 among the 3-year-olds (range: 1.68–2.50), and 2.38 among the 4-year-olds (range: 1.47–2.58).

The median proportion value of the *Metalinguistic awareness* scale grew from 0.31 among the 2;6-year-olds (range: 0–0.47) to 0.46 among the 3-year-olds (range: 0–0.79) and 0.72 among the 4-year-olds (range: 0.11–0.94). One of the items, however, asking whether the child divides words into syllables (e.g., *ba-de, sko-le, le-ke*), did not get higher proportion values with age.

### 3.6. The relationship between item difficulty and item-total correlation

**Figure 4:**
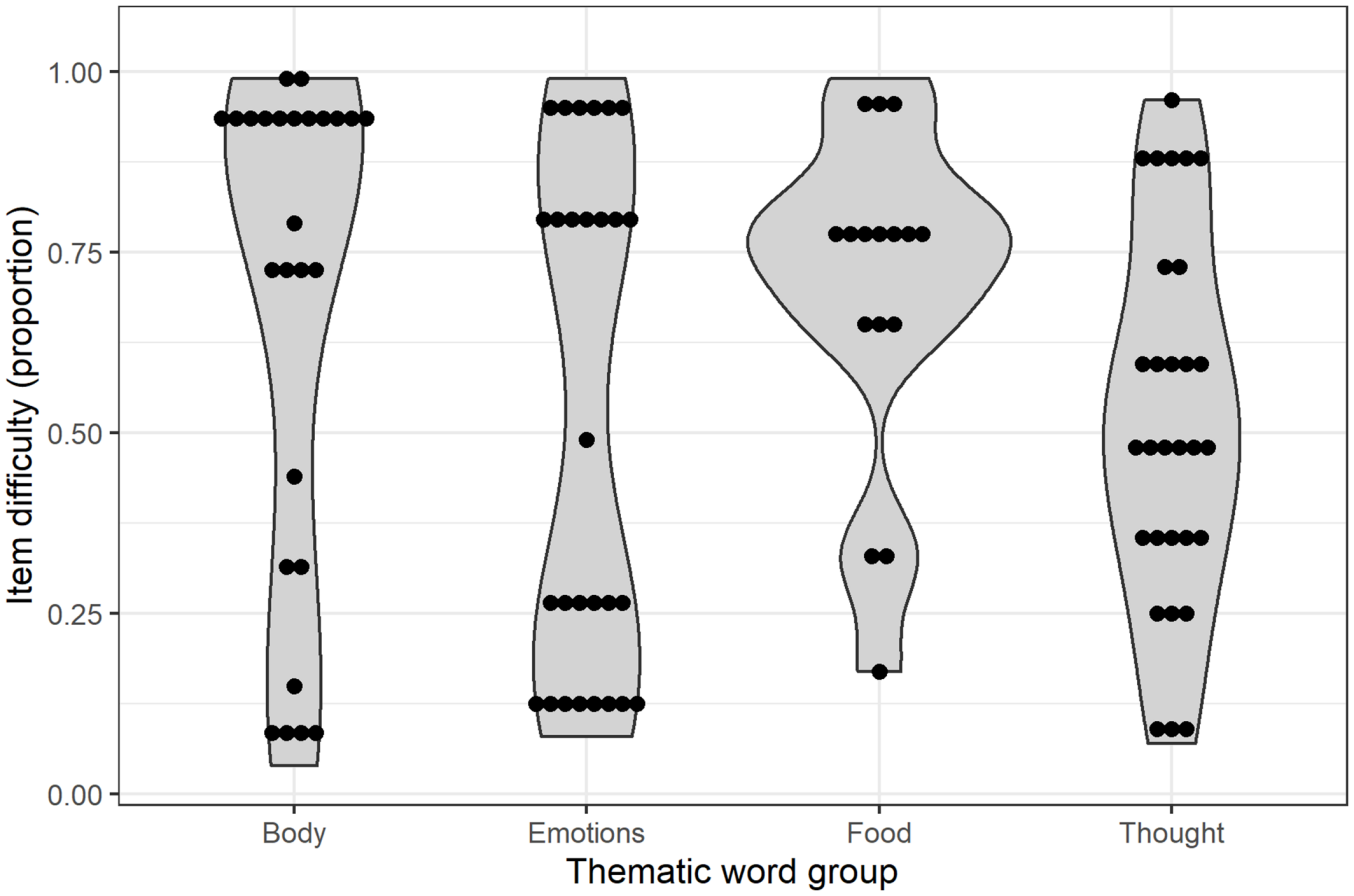
Scatterplot of corrected item-scale correlation coefficients for each word in NCDI-III presented by difficulty (easier items to the right, as they have a higher proportion value).

For the *Vocabulary* section, we found that more words were located at the easy and difficult ends of the scale than near the center, even though the four thematic word groups had differing difficulty profiles. The relationship between item difficulty and corrected item-scale correlation for all the *Vocabulary* items is shown in Figure 4. There is an abundance of very easy items at the right-hand side of the figure. Those are words that were checked by nearly all participants, and consequentially, they tend to have very low corrected item-scale correlation coefficients.

## 4. Discussion

This paper set out to examine psychometric and linguistic properties of the NCDI-III through statistical analyses of data from 100 children. In line with our expectations, we found higher scores with age, an expected gender effect within some scales, strong correlations between the two grammatical scales, and between these and the vocabulary scale. Furthermore, in line with previous research, there was a high internal consistency as measured with Cronbach’s alpha. Regarding dispersion of item difficulty within the vocabulary checklist, we uncovered a bimodal distribution with few words in the mid-difficulty range; globally, within all three age groups and within three of the four thematic word groups. Below, we will discuss our findings in light of previous research, before we elaborate on the bipolar dispersion of difficulty and possible consequences thereof.

### 4.1. Demographics, correlations and consistency

In the *General level of communication* section, all the 4-year-olds, and a majority of the other children, were reported to *often talk in long sentences*, and all the children had a score of 3 or more. This was to be expected, given that no child was included in the study if there was concern about their language development. These results align well with those of (Eriksson, 2017), who reports that 81% of the 1,134 children in the Swedish study “were reported to *talk in long and complicated sentences*” (p. 650), but contrast with findings from Estonian: Only half of Tulviste and Schults’ (2020) 3-year-olds reached max score, compared to 25 of the 28 Norwegian 3-year-olds. This difference is striking, even when we take into account that 20 of the Estonian participants were “described by their parents as experiencing difficulties with language development” (p. 69). There may be several possible explanations for the difference. There could be real differences between languages or between populations. Another possible cause is the slightly differing wording of the most advanced alternative, with ‘long sentences’ in the NCDI-III versus ‘complex sentences’ in the Estonian counterpart. It is possible that parents’ threshold for describing their children’s sentences as ‘complex’ may be higher than the threshold for describing them as ‘long.’

There was growth with age in both of the NCDI-III grammar scales, but the *Grammatical constructions* scale had a considerable ceiling effect both among the 3-year-olds and 4-year-olds. This is in line with Tulviste and Schults’ (2020) findings from Estonian three-year-olds where there was a ceiling effect in the grammatical constructions scale. The SCDI-III grammar scores were merged as a means to resolve issues with ceiling effects (Eriksson, 2017, p. 648). This approach seems reasonable also for Norwegian, but it presupposes weighting two differently scored grammar scales against each other. Whereas the two grammar scales in the Swedish CDI-III have the same answer structure and almost the same maximum scores (20 and 16), the two grammar scales in the Norwegian version differ in answer structures. Consequently, there is a gap in maximum scores, so that the NCDI-III *Sentence complexity* scale’s maximum is 40 while the *Grammatical constructions* scale has a maximum score of only 24. Analyses from an ongoing project on the relationship between vocabulary and grammar in 1–4-year-olds indicate that transforming and merging the two grammar scales resolves ceiling issues also for Norwegian (Holm and Hansen, in progress).

The two items in the NCDI-III *Pronunciation* section also have different measurement scales: The first item’s scale is absolute, asking whether strangers find the child’s speech difficult to understand. As expected, we found growth of age in the answers to this question. The second question factors in age, and thus no growth with age was expected: Parents are asked to *compare the child’s speech to the speech of other children of the same age*. Interestingly, 49 per cent of both Norwegian and Swedish parents report their child to sound like slightly older children, while only 10 per cent in both groups report their child to sound a little younger. Eriksson (2017, p. 652) points out that parental overestimation of their child’s pronunciation may represent a familiarity effect: The parents are used to their own child’s way of speaking, and thus find them easier to understand than other children of the same age.

In line with the Estonian and Swedish results, the Norwegian girls outperformed the boys on *Vocabulary* and *Metalinguistic awareness*. We did however not find any effects of gender on grammar, in contrast to Eriksson (2017). A recent review on gender effects in early language acquisition suggests that gender differences may differ across ages and language domains (Rinaldi et al., 2021); to determine whether gender effects differ between lexical and grammatical knowledge in Norwegian-speaking children, a larger sample might be needed. Like Eriksson (2017) found for the SCDI-III, both birth order and parental education level predicted *Metalinguistic awareness,* with firstborns outperforming laterborns and children with higher-educated parents scoring higher than children with lower-educated parents. *Metalinguistic awareness* thus seems to be sensitive to all demographic variables studied here. Given the skewness toward higher education in the dataset, we cannot conclude that parental education level does not have any influence on the other scales – only that these scales do not appear to distinguish between children of parents with more and less than 3 years of higher education.

Assuming that language skills consist of several different types of abilities where some are more closely interrelated than others, we should expect some parts of the NCDI-III to be more strongly intercorrelated than others. In line with Eriksson (2017) and Tulviste and Schults (2020) as well as a vast literature on younger children, we expected a stronger correlation between vocabulary and grammar. Our findings met our expectations: No correlations were stronger than those between *Vocabulary* and the two grammar scales, and there were only weak correlations between *Pronunciation* and *Vocabulary*, as well as between *Pronunciation* and *Metalinguistic awareness*.

All NCDI-III scales had Cronbach’s alpha values within Eriksson’s (2017) suggested adequacy threshold of >0.65, although *Metalinguistic* awareness only barely so. As the alpha is affected by length, the very high *Vocabulary* score (0.97) could be at least partially attributed to the fact that it is very long (100 items). While a high alpha score is often considered ‘excellent’ and not discussed further, Streiner (2003) points out that a very high alpha could be a sign of redundancy. A different picture appeared when investigating internal consistency through item-scale correlations: In terms of corrected item-scale correlations, there is lower internal consistency within *Vocabulary* than within *Grammatical constructions* and *Sentence complexity*, even if *Vocabulary* had by far the highest alpha.

The *Metalinguistic awareness* section showed a relatively low degree of internal consistency, both in terms of Cronbach’s alpha and in terms of corrected item-total correlations. Eriksson (2017) points out that his results suggest that the *Metalinguistic awareness* scale “taps into a slightly different set of knowledge” than the vocabulary and grammar scales (p.652). This holds also for our Norwegian results, as the scale shows a limited correlation with other sections and is the only scale predicted by all demographic variables in our model. Furthermore, the lower internal consistency suggests that the *Metalinguistic awareness* section is better treated as an assembly of useful questions than as a scale. Each question may still give valuable information about a child’s metalinguistic and pre-literacy development.

### 4.2. Difficulty dispersion

The difference noted for *Vocabulary* between the two consistency measures is connected to its difficulty distribution. As the *Vocabulary* list is fixed while children’s vocabularies grow with age, the word list needs to include both words that are easy enough to distinguish between the youngest children and words that are sufficiently difficult to distinguish between the 4-year-olds. There is thus an inevitable tradeoff between each item’s overall discriminatory power and the total scale’s ability to capture variance across the whole age span. Very easy and very difficult items will necessarily have a low response variance and thereby a low overall discriminatory effect and weak item-total correlation. As pointed out by deVellis (2017, p. 143), “an item that does not vary cannot covary.” Hence, some redundancy in the scale was expected, especially among the youngest and oldest children. More surprising was the overall bimodal difficulty distribution, with few intermediately difficult words both within and across age groups, and a large number of items near the poles of the difficulty continuum. Particularly in the ‘easy’ end of the range, there were many words with a very weak item-scale correlation.

The high Cronbach’s alpha combined with the low consistency in terms of item-scale correlations suggest that the *Vocabulary* scale may be unnecessarily long, and that excessive words could be removed from the easy end of the scale without damaging the instrument’s ability to distinguish between children. Alternatively, one could remove some of the easiest and maybe also some of the hardest words and replace them with words in the medium difficulty range. However, as the sample of participants in this study was skewed toward higher levels of education, and as children whose caregivers were concerned about their linguistic development were excluded from the sample, these tendencies might not be as strong in a more representative sample. A recent response to the issue of redundancy in CDI word lists is the development of adaptive versions based on existing CDI data. Here, parents respond to a dynamic word list that adjusts to their responses, meaning that the researcher achieves the wanted information about each child through far fewer questions (e.g., Mayor and Mani, 2019; Kachergis et al., 2022; Mieszkowska et al., 2022). Such adaptive versions are built on data on large numbers of words from a substantial pool of children. Static forms such as NCDI-III, despite their higher level of redundancy, still offer the advantage of being less resource-intensive in development and standardization, and may be administered without access to digital technology.

The most frequent words in a given language are the ones that tend to be acquired earlier. These constitute a much smaller set of words than the vast amount of less frequent words. As the NCDI-III *Vocabulary* scale is meant to be a proxy measure for a continuous underlying construct – children’s vocabulary size – this poses a challenge: Each of the more ‘sophisticated’ words would generally be sampled from larger and more diverse possible vocabularies than the easy ones, making the use or lack of use of any specific difficult word less representative of a child’s total vocabulary. The NCDI-III follows Eriksson’s (2017) response to this issue, sampling words from a smaller set of topics based on developmental literature. Our finding that the NCDI-III’s body words and food words generally tended to be easier than the words related to thoughts and feelings agrees well with his assumptions. Further, when it comes to body words, most of the NCDI-III words for internal body parts were found among the difficult words.

### 4.3. Limitations

Ideally, a validation or norming sample should resemble the population for which the instrument is meant to be used, and recruitment methods and criteria for inclusion of participants in validation and norming studies thus have consequences for the appropriateness of a tool (see, e.g., Friberg, 2010) In the current study, there is an unusually large overrepresentation of children from families with high education levels. Furthermore, children about whom language concerns had been expressed were left out of the study entirely. These biases may influence our results: The fact that parental education did not appear as a significant predictor of children’s results in most of the NCDI-III scales should not be taken as evidence that parental education cannot predict NCDI-III scores in the general population. To see how well the NCDI-III captures the full breadth of variation found in the Norwegian child population from 2;6 to 4 years, will require further research with a wider selection of participants. Our conclusions regarding the distribution of item difficulty must also be made with the caveat that levels of difficulty calculated from a more representative sample of participants may differ somewhat from what we present in this study.

In the current study, Cronbach’s alpha is used as a measure of the internal consistency –and thereby reliability – of the NCDI-III scales. However, Cronbach’s alpha alone does not give us the *dimensionality* of a scale. It is perfectly possible for a scale measuring two or more underlying constructs to obtain a high alpha, especially if the scale has many items (Streiner, 2003). In order to establish the dimensionality of the NCDI-III scales, a larger study with more participants will be required. However, as Norwegian and Swedish are very closely related languages, and as the Norwegian *Vocabulary* scale is closely modeled on the Swedish one, there is reason to believe that its dimensionality is close to what Eriksson (2017) found for the Swedish adaptation.

The CDI III has so far focused on monolingual children in Western, industrialized, rich and democratic countries. Data from other populations are necessary to assess if generalizations about children’s language acquisition using parental reports with children between 2;6 and 4 are valid also outside of the WEIRD context (Henrich et al., 2010).

### 4.4. Summary

In this paper, we have evaluated the NCDI-III based on a sample of monolingual children between 2;6 and 4 years of age, finding psychometric properties quite similar to what Eriksson (2017) and Tulviste and Schults (2020) have reported for Swedish and Estonian respectively: There is growth with age, and girls outperform boys in the *Vocabulary* and *Metalinguistic awareness* results. There was adequate internal consistency within all scales in terms of Cronbach’s alpha, although less so for *Metalinguistic awareness*. Ceiling effects in the grammar scales could possibly be amended by merging the two scales, but one would have to decide on how to weigh the items from each grammatical scale against the other.

Based on findings from our analyses of item difficulty distribution and internal consistency, we suggest that subsequent CDI-III adaptations may benefit from paying attention to the difficulty profiles of the scales, preferably avoiding items too close to the poles of the difficulty range.

## Data availability statement

The dataset underlying the analyses in this article will be made available through the Wordbank database (http://wordbank.stanford.edu/data).

## Ethics statement

The studies involving human participants were reviewed and approved by the Norwegian Data Protection Services, SIKT (Norwegian Agency for Shared Services in Education and Research, formerly NSD, Norwegian Centre for Research Data) under the number 788858. Written informed consent to participate in this study was provided by the participants’ legal guardian/next of kin.

## Author contributions

NG and AR had the idea for an evaluation of the NCDI-III. EH was recruited to carry out the study. The data collection was initiated by the research project at OsloMet, led by NG with AR and PH as close collaborators, and carried out by master’s students. EH organized the last round of data collection, and proposed and conducted the analyses of item difficulty profiles, along with using Tobit regression for the modeling of relationships between NCDI-III results and demographic factors. EH, PH, and AR wrote summaries of background literature for the other co-authors. EH prepared and carried out the statistical analyses, discussing them with the others, especially PH, in the process. PH contributed substantially to the analyses, with practical knowledge and in discussions of methods, interpretations of results, and preparation of figures and tables. EH drafted most of the article text, but all co-authors have drafted paragraphs. All co-authors have discussed and revised the text in collaboration, in several rounds. All authors have suggested literature, participated in discussions about possible designs for the manuscript and research questions, and read through the final draft of the article before submitting it for peer review.

## Funding

The research has been mainly funded by Faculty of Education and International Studies (LUI), OsloMet - Oslo Metropolitan University, and publishing is funded by Department of Research and Development, OsloMet - Oslo Metropolitan University.

## Conflict of interest

The authors declare that the research was conducted in the absence of any commercial or financial relationships that could be construed as a potential conflict of interest.

## Publisher’s note

All claims expressed in this article are solely those of the authors and do not necessarily represent those of their affiliated organizations, or those of the publisher, the editors and the reviewers. Any product that may be evaluated in this article, or claim that may be made by its manufacturer, is not guaranteed or endorsed by the publisher.

## Editor’s note

Maria-José Ezeizabarrena edited the article in collaboration with Melita Kovacevic, University of Zagreb, Zagreb, Croatia.

## References

[ref1] Bleses D. Vach W. Slott M. Wehberg S. Thomsen P. Madsen T. O.. (2008). Early vocabulary development in Danish and other languages: a CDI-based comparison. J. Child Lang. 35, 619–650. doi: 10.1017/S0305000908008714, PMID: 18588717

[ref2] Charman T. Drew A. Baird C. Baird G. (2003). Measuring early language development in preschool children with autism spectrum disorder using the MacArthur communicative development inventory (infant form). J. Child Lang. 30, 213–236. doi: 10.1017/s0305000902005482, PMID: 12718299

[ref3] DeVellis R. F. (2017). Scale development: Theory and applications, vol. 26. 4th *Edn*. Los Angeles: SAGE.

[ref4] Dionne G. Dale P. S. Boivin M. Plomin R. (2003). Genetic evidence for bidirectional effects of early lexical and grammatical development. Child Dev. 74, 394–412. doi: 10.1111/1467-8624.7402005, PMID: 12705562

[ref5] Eriksson M. (2017). The Swedish communicative development inventory III: parent reports on language in preschool children. Int. J. Behav. Dev. 41, 647–654. doi: 10.1177/0165025416644078, PMID: 28890587 PMC5574490

[ref6] Eriksson M. Marschik P. B. Tulviste T. Almgren M. Pérez Pereira M. Wehberg S.. (2012). Differences between girls and boys in emerging language skills: evidence from 10 language communities. Br. J. Dev. Psychol. 30, 326–343. doi: 10.1111/j.2044-835X.2011.02042.x 22550951

[ref7] Ezeizabarrena M.-J. Barnes J. García I. Barreña A. Almgren M. (2013). “Using parental report assessment for bilingual preschoolers: the Basque experience” in Solutions for the assessment of bilinguals. ed. Gathercole V. C. M. doi: 10.21832/9781783090150

[ref8] Feldman H. M. Campbell T. F. Kurs-Lasky M. Rockette H. E. Dale P. S. Colborn D. K.. (2005). Concurrent and predictive validity of parent reports of child language at ages 2 and 3 years. Child Dev. 76, 856–868. doi: 10.1111/j.1467-8624.2005.00882.x, PMID: 16026501 PMC1350485

[ref9] Fenson L. Dale P. S. Reznick J. S. Bates E. Thal D. J. Pethick S. J. (1994). Variability in early communicative development. Monogr. Soc. Res. Child Dev. 59:i. doi: 10.2307/1166093 7845413

[ref10] Fenson L. Marchman V. A. Thal D. J. Dale P. S. Reznick J. S. Bates E. (2007). MacArthur-Bates communicative development inventories. Baltimore, MD: Paul H. Brookes Publishing Company.

[ref11] Flygstad N. Milder N. (2017). Utprøving av CDI-III for norske enspråklige treåringer: En pilotstudie. Master’s thesis, University of Oslo.

[ref12] Frank M. C. Braginsky M. Yurovsky D. Marchman V. A. (2017). Wordbank: an open repository for developmental vocabulary data. J. Child Lang. 44, 677–694. doi: 10.1017/S0305000916000209, PMID: 27189114

[ref13] Frank M. C. Braginsky M. Yurovsky D. Marchman V. A. (2021). Variability and consistency in early language learning: The Wordbank project. Cambridge, MA: MIT Press.

[ref14] Friberg J. C. (2010). Considerations for test selection: how do validity and reliability impact diagnostic decisions? Child Lang Teach Ther 26, 77–92. eric. doi: 10.1177/0265659009349972

[ref15] Garcia I. Barreña A. Ezeizabarrena M.-J. Almgren M. Arratibel N. Barnes J. (2014). Haur euskaldunen komunikazio-garapena neurtzen 30-50 hilabete bitartean: MacArthur-Bates CDI-III tresnaren euskal bertsioa [assessing the communicative development of 30 to 50-month old Basque children: the Basque version of the MacArthur-Bates CDI-III]. Uztaro 88, 33–72. doi: 10.26876/uztaro.88.2014.3

[ref16] Garmann N. G. Romøren A. S. H. Flygstad N. Milder N. Pedersen I. Simonsen H.. (2019). Språkkartlegging i norske barnehager - en pilotundersøkelse av foreldres og barnehageansattes rapportering av norsktalende tre-åringers språkferdigheter ved hjelp av CDI III. RASK: Internationalt Tidsskrift for Sprog Og Kommunikation, 49, 87–103.

[ref17] Guiberson M. (2008). Validity of a parent vocabulary checklist for young Spanish speaking children of Mexican immigrants. Int. J. Speech Lang. Pathol. 10, 279–285. doi: 10.1080/17549500802216763, PMID: 20840027

[ref18] Heilmann J. Ellis Weismer S. Evans J. Hollar C. (2005). Utility of the MacArthur-Bates communicative development inventory in identifying language abilities of late-talking and typically developing toddlers. Am. J. Speech Lang. Pathol. 14, 40–51. doi: 10.1044/1058-0360(2005/006), PMID: 15966111

[ref19] Henrich J. Heine S. J. Norenzayan A. (2010). The weirdest people in the world? Behav. Brain Sci. 33, 61–83. doi: 10.1017/S0140525X0999152X, PMID: 20550733

[ref20] Holm E. Hansen P. (in progress). The relationship between vocabulary and grammar in Norwegian 1;6-4-year-olds (manuscript in preparation).

[ref21] Jackson-Maldonado D. Marchman V. A. Dale P. Rubio-Codina M. (2022). The MacArthur-Bates CDI-III for Spanish-speaking children.

[ref22] Jackson-Maldonado D. Marchman V. A. Fernald L. C. H. (2013). Short-form versions of the Spanish MacArthur–Bates communicative development inventories. Appl. Psycholinguist. 34, 837–868. doi: 10.1017/S0142716412000045

[ref23] Kachergis G. Marchman V. A. Dale P. S. Mankewitz J. Frank M. C. (2022). Online computerized adaptive tests of Children’s vocabulary development in English and Mexican Spanish. J. Speech Lang. Hear. Res. 65, 2288–2308. doi: 10.1044/2022_JSLHR-21-00372, PMID: 35658517 PMC9567402

[ref24] Kas B. Jakab Z. Lőrik J. (2022). Development and norming of the Hungarian CDI-III: a screening tool for language delay. Int. J. Lang. Commun. Disord. 57, 252–273. doi: 10.1111/1460-6984.12686, PMID: 34997807 PMC9304143

[ref25] Kleiber C. Zeileis A. (2008). Applied econometrics with {R}. Springer-Verlag. Available at: https://CRAN.R-project.org/package=AER

[ref26] Kristoffersen K. E. Simonsen H. G. Eiesland E. A. Henriksen L. Y. (2012). Utvikling og variasjon i kommunikative ferdigheter hos barn som lærer norsk – en CDI-basert studie. Norsk Tidsskrift Logopedi 58, 34–43.

[ref27] Law J. Roy P. (2008). Parental report of infant language skills: a review of the development and application of the communicative development inventories. Child Adolesc. Mental Health 13, 198–206. doi: 10.1111/j.1475-3588.2008.00503.x, PMID: 32847184

[ref28] Mayor J. Mani N. (2019). A short version of the MacArthur–Bates communicative development inventories with high validity. Behav. Res. Methods 51, 2248–2255. doi: 10.3758/s13428-018-1146-0, PMID: 30306410

[ref29] Mieszkowska K. Krajewski G. Król P. Krysztofiak M. Haman E. (2022). Developing adaptive CDIs in polish and the CDI-online web app. In Proceeding of EUNM-CDI 2022: The eighth European network meeting on communicative development inventories, Dubrovnik, Croatia.

[ref30] R Core Team. (2021). R: A language and environment for statistical computing [manual]. R Foundation for Statistical Computing. Available at: https://www.R-project.org/

[ref31] Revelle W. (2021). Psych: procedures for psychological, psychometric, and personality research. Northwestern University. Available at: https://CRAN.R-project.org/package=psych

[ref32] Rinaldi P. Pasqualetti P. Volterra V. Caselli M. C. (2021). Gender differences in early stages of language development. Some evidence and possible explanations. J. Neurosci. Res. 101, 643–653. doi: 10.1002/jnr.24914, PMID: 34240751

[ref33] RStudio Team. (2021). RStudio: Integrated development environment for R [manual]. RStudio, PBC. Available at: http://www.rstudio.com/

[ref34] Scherer N. D’Antonio L. (1995). Parent questionnaire for screening early language development in children with cleft palate. Cleft Palate Craniofacial J 32, 7–13. doi: 10.1597/1545-1569(1995)032<0007:PQFSEL>2.3.CO;2, PMID: 7727490

[ref35] Statistics Norway. (2022). Educational attainment of the population. Available at: https://www.ssb.no/en/utdanning/utdanningsniva/statistikk/befolkningens-utdanningsniva

[ref36] Stolt S. (2023). Internal consistency and concurrent validity of the parental report instrument on language in pre-school-aged children – The Finnish Communicative Development Inventory III. First Language, 01427237231167301. doi: 10.1177/01427237231167301

[ref37] Streiner D. L. (2003). Starting at the beginning: an introduction to coefficient alpha and internal consistency. J. Pers. Assess. 80, 99–103. doi: 10.1207/S15327752JPA8001_18, PMID: 12584072

[ref38] Sunde J. A. Erikstad N. H. Andersen K. (2014). Validering av MacArthur-Bates Communicative Development Inventories III [CDI-III] for norsk enspråklige og engelsk-norsk tospråklige barn mellom tre og fire år – En pilotstudie Master’s thesis. Bergen: University of Bergen.

[ref39] Thal D. DesJardin J. L. Eisenberg L. S. (2007). Validity of the MacArthur-Bates communicative development inventories for measuring language abilities in children with Cochlear implants. Am. J. Speech Lang. Pathol. 16, 54–64. doi: 10.1044/1058-0360(2007/007), PMID: 17329675

[ref40] Tobin J. (1958). Estimation of relationships for limited dependent variables. Econometrica 26, 24–36. doi: 10.2307/1907382

[ref41] Tulviste T. Schults A. (2020). Parental reports of communicative development at the age of 36 months: The Estonian CDI-III. First Language, 40, 64–83. doi: 10.1177/0142723719887313

[ref42] Urm A. Tulviste T. (2021). Toddlers’ early communicative skills as assessed by the short form version of the Estonian MacArthur-Bates communicative development inventory II. J. Speech Lang. Hear. Res. 64, 1303–1315. doi: 10.1044/2020_JSLHR-20-00201, PMID: 33755517

[ref43] Wehberg S. Vach W. Bleses D. Thomsen P. Madsen T. O. Basbøll H. (2008). Girls talk about dolls and boys about cars? Analyses of group and individual variation in Danish children’s first words. First Lang. 28, 71–85. doi: 10.1177/0142723707081729

[ref01] Wei T. Simko V. (2021). R package ‘corrplot’: visualization of a correlation matrix. Available at: https://github.com/taiyun/corrplot

[ref44] Wickham H. (2016). ggplot2: elegant graphics for data analysis. Available at: https://ggplot2.tidyverse.org

[ref45] Wickham H. François R. Henry L. Müller K. (2021). dplyr: a grammar of data manipulation. Available at: https://CRAN.R-project.org/package=dplyr

