# Supplementary material for: The Norwegian CDI-III as an assessment tool for lexical and grammatical development in preschoolers
Source: Front Psychol. 2023 Jul 24;14:1175658. doi: 10.3389/fpsyg.2023.1175658 (PMC10408306; doi:10.3389/fpsyg.2023.1175658)
Supplement: Supplementary file 1 [file Presentation_1.pdf]

Appendix

Intercorrelations between NCDI-III sections as calculated using Pearson's product-moment correlation, Spearman's rank correlation rho, and Kendall' rank correlation tau

The correlation plots below were made using the *corrplot* package version 0.92 (Wei and Simko, 2021).

Figure A1

Intercorrelations among the sections of NCDI-III as calculated using Pearson's product-moment correlation

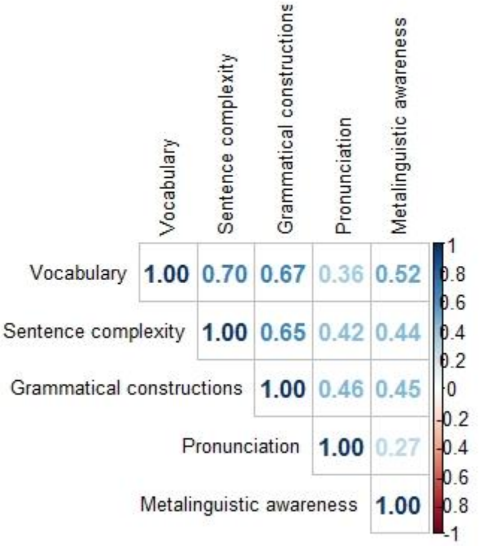

Figure A2

Intercorrelations among the sections of NCDI-III as calculated using Spearman's rank correlation rho

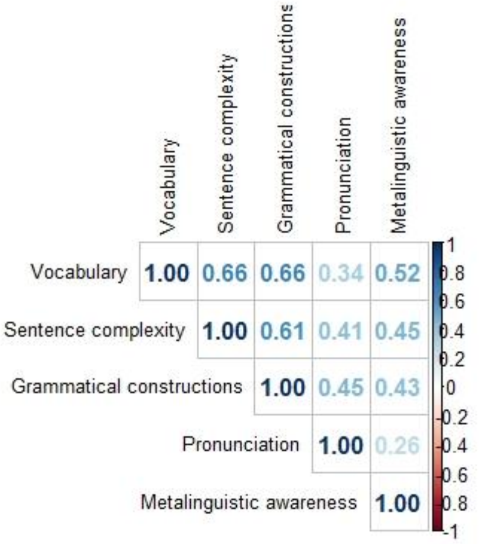

Figure A3

Intercorrelations among the sections of NCDI-III as calculated using Kendall' rank correlation tau

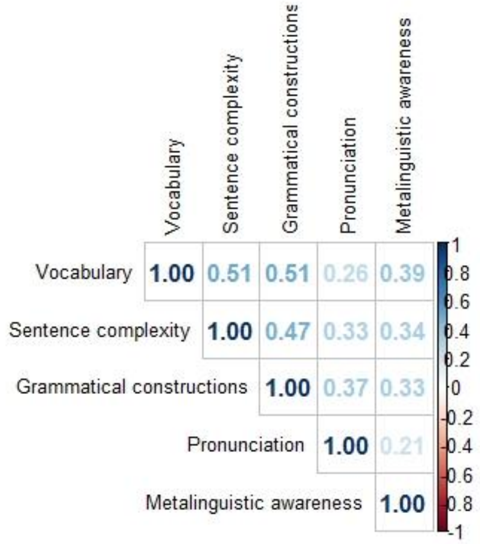

Reference

Wei, T. and Simko, V. (2021). R package 'corrplot': visualization of a correlation matrix. Available at: https://github.com/taiyun/corrplot
